# Immunological effects of supervised exercise in long COVID in adults: A secondary analysis of a randomized crossover trial

**DOI:** 10.14814/phy2.71031

**Published:** 2026-08-28

**Authors:** Nora García‐Alonso, Mikel Izquierdo, Hugo Arasanz, Robinson Ramírez‐Vélez

**Affiliations:** ^1^ Instituto de Investigación Sanitaria de Navarra (IdiSNA) Navarrabiomed, Hospital Universitario de Navarra (HUN), Universidad Pública de Navarra (UPNA) Pamplona Spain; ^2^ CIBER of Frailty and Healthy Aging (CIBERFES) Instituto de Salud Carlos III Madrid Spain; ^3^ Medical Oncology Department Hospital Universitario de Navarra Pamplona Spain; ^4^ Oncobiona Group, Navarrabiomed Pamplona Spain; ^5^ IdiSNA; Oncoimmunology Group Pamplona Spain

**Keywords:** cytokines, exercise training, immunophenotyping, long COVID, T lymphocytes

## Abstract

Long COVID (post‐acute sequelae of SARS‐CoV‐2 infection) affects approximately 5%–30% of survivors and is characterized by persistent fatigue, dyspnea, exercise intolerance, and cognitive impairment. We evaluated the immunological effects of supervised exercise in adults with long COVID. In this pre‐specified exploratory substudy of the EXER‐COVID randomized 2 × 2 crossover trial, participants completed a 6‐week supervised exercise program (twice weekly) or usual care before crossing over after a 3–5 day washout. Plasma cytokines (IL‐1β, IL‐6, IL‐10, TNF‐α, MCP‐1/CCL2, MIP‐1α/CCL3, MIP‐1β/CCL4, and IP‐10/CXCL10) were measured by multiplex immunoassay. Immunophenotyping included 20 CD4^+^ and CD8^+^ T‐cell subsets and five innate immune populations. Treatment effects were estimated using within‐period change scores, with multiple testing controlled by the Benjamini–Hochberg procedure (false discovery rate < 5%). Five variables reached nominal significance before correction (two CD8^+^ T‐cell subsets and IL‐1β, IL‐10, and MIP‐1α), but none remained significant after adjustment. No changes were observed in innate immune populations, and no evidence of carryover was detected. Supervised exercise was not associated with significant immunological changes after correction for multiple comparisons, supporting the short‐term immunological safety of moderate‐intensity exercise in PESE‐negative adults with long COVID (ClinicalTrials.gov: NCT04797871).

## 1 INTRODUCTION

Long COVID—defined as post‐acute sequelae of SARS‐CoV‐2 infection (PASC)—affects an estimated 5%–30% of survivors and is characterized by persistent fatigue, dyspnea, exercise intolerance, and cognitive impairment (Nalbandian et al., 2021; Xie et al., 2024). These symptoms substantially impair health‐related quality of life; yet few disease‐modifying therapies have been established. Exercise intolerance in long COVID is multifactorial, involving skeletal muscle deconditioning, impaired peripheral oxygen extraction, autonomic dysfunction, and ventilatory abnormalities (Durstenfeld et al., 2022) it does not respond consistently to pharmacological intervention (Seo et al., 2025). Structured exercise training reliably improves exercise tolerance in other chronic conditions—including heart failure and post‐viral fatigue syndromes—and has been proposed as a component of rehabilitation following severe respiratory infection (Gloeckl et al., 2024; National Institute for Health and Care Excellence, 2020).

Current consensus guidelines acknowledge a potential role for exercise rehabilitation while emphasizing caution, particularly in patients with post‐exertional symptom exacerbation (PESE) (Gloeckl et al., 2024). The National Institute for Health and Care Excellence (NICE) recommends that exercise be individualized and withheld when it precipitates severe PESE (National Institute for Health and Care Excellence, 2020). Practical frameworks distinguishing patients with and without clinically significant PESE generally advocate low‐to‐moderate intensity endurance or combined training delivered under supervision, and early data suggest that such protocols can safely improve functional status (National Institute for Health and Care Excellence, 2020). Randomized controlled trials indicate that supervised exercise modestly improves aerobic capacity and patient‐reported outcomes in long COVID (Ramírez‐Vélez et al., 2024). In a Spanish trial enrolling 39 adults with symptoms persisting for more than 12 weeks, 8 weeks of twice‐weekly supervised concurrent aerobic and resistance training increased peak oxygen consumption (V̇O_2_peak) by approximately 5.7% relative to usual care, with concurrent improvements in muscle strength, fatigue, depression, and health‐related quality of life (Jimeno‐Almazán et al., 2022).

Meta‐analytic data from cardiopulmonary exercise testing (CPET) demonstrate that persons with long COVID exhibit V̇O_2_peak values approximately 5.0 mL·kg^−1^·min^−1^ lower than those of recovered controls (Durstenfeld et al., 2022); a deficit that exceeds the minimal clinically important difference of approximately 1.0 mL·kg^−1^·min^−1^ typically cited in pulmonary rehabilitation, a threshold met or approached in the supervised trials noted above. Benefit is not universal, however: a large multicentre trial of daily home‐based resistance exercise found no significant improvement in symptoms, exercise capacity, or well‐being (Berry et al., 2025), underscoring the importance of intervention intensity, supervision, and patient selection.

The immunomodulatory effects of exercise in long COVID remain incompletely characterized. SARS‐CoV‐2 infection provokes profound systemic inflammation, and persistent immune dysregulation—including alterations in interferon signaling, T‐cell phenotype, and circulating cytokine profiles—has been documented in PASC and proposed to contribute to its cardinal symptoms (Durstenfeld et al., 2022; Nalbandian et al., 2021; National Institute for Health and Care Excellence, 2020). In healthy individuals, acute exercise transiently mobilizes lymphocytes and elevates circulating cytokines, and regular training attenuates chronic low‐grade inflammation across several clinical populations. Evidence from post‐COVID cohorts is inconsistent. In one trial of COVID‐19 survivors, 12 weeks of supervised training reduced concentrations of monocyte chemoattractant protein‐1 (MCP‐1/CCL2) and interferon‐γ and increased interleukin‐10 (IL‐10) (Filgueira et al., 2023). These findings are broadly consistent with those of (Abbasi et al., 2024) who conducted a single‐arm pilot study involving 14 individuals with long COVID. After 10 weeks of supervised aerobic exercise, participants demonstrated improvements in cardiorespiratory fitness and fatigue, whereas no significant changes were observed in circulating cytokines or leukocyte subsets. These discrepant findings may reflect heterogeneity in training protocols, patient populations, and analytical approaches, as well as the inherent complexity of immune responses in a condition characterized by heterogeneous immunological trajectories.

Systematic characterization of the immunological response to exercise in long COVID requires simultaneous assessment of soluble mediators and cellular immune compartments. Measuring these two domains in parallel allows determination of whether any exercise‐associated immune changes are global or restricted to specific compartments—a distinction with direct implications for understanding the mechanism and for selecting appropriate endpoints in future trials. The present study used a pre‐specified, randomized 2 × 2 crossover design to conduct this integrated assessment in individuals with long COVID, with biological context provided by pathway annotation of the cytokine panel.

## 2 METHODS

### 2.1 Trial oversight and study design

The immunological outcomes reported here were pre‐specified as secondary endpoints of the EXER‐COVID trial, a randomized, single‐centre, 2 × 2 AB/BA crossover study in adults with long COVID (ClinicalTrials.gov: NCT04797871, Version 2, registered 15 March 2021). The primary outcomes and main intervention effects have been reported previously (Ramírez‐Vélez et al., 2024). The present analysis reports the pre‐specified immunological secondary outcomes and is conducted and reported in accordance with the CONSORT extension for secondary analyses of randomized trials. The study was conducted in accordance with the Declaration of Helsinki, and the protocol was approved by the Research Ethics Board of Hospital Universitario de Navarra (reference: PI_2020/140), and all participants provided written informed consent before enrolment.

Participants were randomly assigned to one of two treatment sequences: exercise followed by usual care (sequence AB) or usual care followed by exercise (sequence BA). The two 6‐week intervention periods were separated by a 3–5 days washout interval. This interval was selected to balance participant burden against study feasibility. For cytokine endpoints, acute exercise‐induced perturbations in circulating interleukins and chemokines typically resolve within 24–72 h in most populations (Peake et al., 2017); accordingly, a 3–5 day washout is likely sufficient; for cellular immunophenotypes—in particular memory and senescence‐associated CD8^+^ T‐cell subsets, which can undergo durable phenotypic and transcriptional remodeling persisting over weeks to months—this washout is insufficient to guarantee full phenotypic reversal. Carryover effects in CD8^+^ outcomes are therefore expected by design and should be considered a pre‐specified interpretive caveat for cellular endpoints rather than an unexpected finding.

The crossover design was selected to improve statistical efficiency by enabling within‐person comparisons, thereby reducing between‐subject variability. Assessments were conducted at baseline (T1), at the end of period 1 (T2), and at the end of period 2 (T3). The primary analytic quantity for each treatment period was the within‐period change score (end‐of‐period minus pre‐period value). This approach accommodated the repeated‐measures data structure while permitting estimation of treatment, period, and sequence effects.

### 2.2 Participants and randomization

Adults meeting established criteria for long COVID were enrolled in the EXER‐COVID trial. Cytokine data were available for 36 participants distributed across the two randomized sequences (sequence AB: *n* = 17; sequence BA: *n* = 19). A subsample of 35 participants had peripheral blood mononuclear cell (PBMC) data available for exploratory lymphocyte immunophenotyping. Randomization was performed using a web‐based block randomization procedure with a 1:1 allocation ratio. Investigators responsible for laboratory measurement and statistical analysis were blinded to treatment allocation where feasible; blinding of participants and exercise personnel was not possible given the nature of the intervention.

### 2.3 Interventions

The exercise intervention consisted of a 6‐week supervised power resistance training exercise program comprising two sessions per week on nonconsecutive days to prevent overtraining and to maximize adaptations, delivered according to published rehabilitation for post‐COVID‐19 condition (Berry et al., 2025; Jimeno‐Almazán et al., 2022). The duration of the sessions was 45–50 min, and the duration of the entire program was 6 weeks, equivalent to 12 sessions. Mean session attendance was 91.7% (11 of 12 sessions; full details reported in the primary trial publication (Ramírez‐Vélez et al., 2024)). Participants assigned to sequence AB received the exercise intervention during period 1 and usual care during period 2; those assigned to sequence BA received usual care first and the exercise intervention second. Before each training session, participants were screened for worsening post‐exertional symptoms. Participants were instructed to refrain from any other structured exercise or rehabilitation program during the trial. Usual care followed standard clinical recommendations and did not include systematic exercise or supervised pulmonary rehabilitation.

#### 2.3.1 Clinical outcomes

Baseline demographic and clinical characteristics were collected using a standardized questionnaire at study enrollment. Variables included sex, age, marital status, educational attainment, employment status, vaccination status, and medication use. Sex was recorded as female or male. Age was summarized as a continuous variable and reported as mean (SD). Marital status was categorized as married or not married. Educational attainment was classified as college graduate or higher versus lower levels. Employment status was defined as full‐time employment versus not employed full time.

Vaccination status at enrollment was categorized as receipt of 2 or more doses, receipt of a booster dose, or unknown status. Medication use was assessed as current use of any medication (yes or no), and participants were further classified as reporting any medication use or none. Peak oxygen consumption (peak V̇O_2_) was assessed at baseline using cardiopulmonary exercise testing. Lactate thresholds were not measured; however, the first (VT1) and second (VT2) ventilatory thresholds were analyzed. These are widely used and validated functional indicators in clinical exercise studies, providing a reliable estimate of the metabolic transition during gradual exertion (Ramírez‐Vélez et al., 2024). Muscle strength was evaluated through grip strength measurements using a digital Grip‐D hand dynamometer (Takei 5401, Tokyo, Japan), and dynamic maximal muscle strength was determined using a one‐repetition maximum (1RM) test. The 1RM test was conducted for bilateral leg press, knee extension, chest press, and back press exercises, with higher values indicating better overall health and muscle function. Anthropometric measurements, including body weight and height, were obtained using an SECA scale and stadiometer (model 799 Electronic Column Scale, Hamburg, Germany). Body composition parameters, including lean mass, fat mass, and appendicular lean mass, were assessed using dual‐energy X‐ray absorptiometry (HOLOGIC; Discovery Wi, Marlborough, MA, USA).

### 2.4 Immunological outcomes

#### 2.4.1 Lymphocyte immunophenotyping

The cellular immunological assessment encompassed 21 circulating lymphocyte immunophenotypes derived from PBMCs, with emphasis on CD4+ and CD8+ T‐cell subsets. Peripheral blood samples were processed immediately after collection. PBMCs were isolated by density‐gradient centrifugation using Ficoll‐Paque™ PLUS (Cytiva, Uppsala, Sweden; Cat. No. 17‐1440‐02). After centrifugation, the mononuclear cell layer was collected, washed twice with phosphate‐buffered saline (PBS), and prepared for immunophenotyping and stained with fluorochrome‐conjugated antibodies against 21 T‐lymphocyte subpopulations characterized by surface expression of CD4, CD8, CD28, CD62L, CD57, KLRG1, and PD‐1 (lymphocyte panel), and five innate immune cell populations monocytes, M2‐like monocytes [CD11b^+^CD116^+^CD14^+^CD66b^−^CD163^+^], NK cells, neutrophils, and low‐density neutrophils [LDN; CD11b^+^CD66b^+^CD116^+^CD119^−^] All AI‐assisted text was critically reviewed, revised, and approved by the authors, who take full responsibility for the manuscript's content., constituting the innate immune panel. The CD163^+^ population is referred to as “M2‐like monocytes” to distinguish it from tissue‐resident M2 macrophages, which are not reliably recovered from peripheral blood. The LDN population co‐purifies with PBMCs during density‐gradient centrifugation and is phenotypically consistent with the low‐density neutrophil fraction documented in inflammatory and post‐viral states. Immune cell subset proportions were quantified by flow cytometry and analyzed using FlowJo software. Cell populations are reported as percentage of parent gate (lymphocyte and innate panels).

#### 2.4.2 Cytokine quantification

Soluble immune mediators measured included interleukin‐1β (IL‐1β), interleukin‐6 (IL‐6), interleukin‐10 (IL‐10), tumor necrosis factor‐α (TNF‐α), monocyte chemoattractant protein‐1 (MCP‐1/CCL2), macrophage inflammatory protein‐1α (MIP‐1α/CCL3), macrophage inflammatory protein‐1β (MIP‐1β/CCL4), and interferon‐γ–inducible protein‐10 (IP‐10/CXCL10). Blood samples were obtained after an overnight fast, at each assessment timepoint (T1, T2, and T3), within 24–72 h of the final training session of each period, processed according to standardized protocols, and plasma was stored at −80°C until analysis. Cytokine concentrations were quantified using the MILLIPLEX® Human Cytokine/Chemokine/Growth Factor Panel (MilliporeSigma, Burlington, MA, USA) on a Luminex xMAP® platform (Luminex Corporation, Austin, TX, USA). Samples were analyzed in duplicate by the same trained operator according to the manufacturer's instructions, and all samples from each participant were assayed within the same analytical batch to minimize inter‐assay variability.

### 2.5 Statistical analysis

All analyses follow the pre‐specified plan for a 2 × 2 AB/BA crossover design. The primary analysis is described first; sensitivity analyses are clearly labeled as secondary. Full Bayesian model specification is provided in Supplementary Methods Analysis. Clinical variables were pre‐specified and analyzed as categorical or continuous variables, as appropriate. Descriptive statistics for lymphocyte subsets, cytokines, and innate immune markers, stratified by treatment group and time point, are provided in Table S1. The primary treatment contrast was the mean difference in change scores between the exercise and standard‐care periods, estimated via a one‐sample *z*‐test on the paired contrasts (exercise change minus standard‐care change). Ninety‐five percent confidence intervals were calculated from the empirical standard error of the contrasts. Differential carryover was assessed by a Welch two‐sample *t*‐test comparing period‐sum scores between sequences (Grizzle test); a period effect was assessed analogously using period‐difference scores. Multiple comparisons across all outcomes (lymphocyte, cytokine, and innate panel) were controlled using the Benjamini‐Hochberg (BH) procedure separately within each panel, with a false discovery rate threshold of 5%. All analyses were performed using R version 4.5.1 (R Foundation for Statistical Computing, Vienna, Austria), with the lme4 package (version 1.1–37) for linear mixed‐effects modeling and the mice package (version 3.18.0) for multiple imputation. Twenty imputed datasets were generated under the assumption that data were missing at random, and parameter estimates were combined using Rubin's rules.

## 3 RESULTS

### 3.1 Baseline characteristics

Baseline characteristics were similar between participants assigned to the exercise‐first (AB) and usual care–first (BA) sequences (Table 1). The mean (SD) age of the cohort was 47.7 (9.7) years, and 63.9% were women. No significant between‐group differences were observed in demographic characteristics, including sex distribution, marital status, educational attainment, employment status, vaccination status, or medication use (all *p* > 0.05).

**TABLE 1:** Baseline characteristics of participants according to randomization sequence.

Characteristic	Exercise first (AB) (*n* = 17)	Usual care first (BA) (*n* = 19)	Total (*n* = 36)	*p*
Demographic characteristics				
Female sex, No. (%)	13 (76.4)	10 (52.6)	23 (63.9)	0.563
Age, mean (SD), y	47.20 (10.09)	48.21 (9.64)	47.73 (9.72)	0.760
Married, No. (%)	9 (52.9)	15 (78.9)	24 (66.7)	0.280
College graduate or higher, No. (%)	14 (82.3)	12 (63.1)	26 (72.2)	0.442
Full‐time employment, No. (%)	11 (64.7)	11 (57.8)	22 (61.1)	1.000
Vaccination status at enrollment, No. (%)				
≥ 2 doses	13 (76.4)	14 (73.6)	27 (75.0)	0.807
Booster dose	2 (11.7)	4 (21.0)	6 (16.7)	
Unknown	2 (11.7)	0 (0)	3 (8.3)	
Medication use, No. (%)				
Any medication use	10 (58.8)	9 (47.3)	19 (52.8)	0.331
None	7 (41.2)	10 (52.7)	17 (47.2)	
Cardiopulmonary parameters				
VT1 V̇O_2_, mean (SD), mL·kg^−1^·min^−1^	8.90 (2.79)	9.04 (1.43)	8.97 (2.15)	0.853
VT2 V̇O_2_, mean (SD), mL·kg^−1^·min^−1^	21.35 (6.72)	20.43 (3.98)	20.86 (5.39)	0.616
Peak V̇O_2_, mean (SD), mL·kg^−1^·min^−1^	23.42 (7.37)	21.56 (4.38)	22.44 (5.97)	0.356
VT1 V̇E, mean (SD), L/min	19.73 (7.01)	20.51 (5.75)	20.14 (6.29)	0.717
VT2 V̇E, mean (SD), L/min	53.46 (19.93)	55.62 (18.35)	54.60 (18.87)	0.737
Peak V̇E, mean (SD), L/min	65.80 (24.98)	65.16 (21.49)	65.46 (22.87)	0.934
V̇E/V̇CO_2_ slope, mean (SD)	32.86 (3.54)	34.44 (5.09)	33.70 (4.44)	0.290
Peak workload, mean (SD), W	133.82 (57.24)	122.37 (39.87)	127.78 (48.47)	0.487
Peak heart rate, mean (SD), bpm	159.35 (18.53)	153.0 (17.07)	156.09 (17.82)	0.299
Muscle strength				
Chest press 1RM, mean (SD), kg	48.11 (18.58)	55.94 (27.90)	51.81 (23.44)	0.324
Leg press 1RM, mean (SD), kg	168.65 (86.0)	171.95 (55.22)	170.39 (70.37)	0.891
Knee extension 1RM, mean (SD), kg	78.65 (40.42)	79.42 (32.75)	79.06 (36.03)	0.950
Back press 1RM, mean (SD), kg	62.29 (31.23)	60.21 (21.93)	61.19 (26.35)	0.817
Grip strength, mean (SD), kg	29.63 (10.38)	27.33 (8.82)	28.42 (9.52)	0.478
Anthropometric and body composition				
Body mass, mean (SD), kg	75.70 (15.70)	75.78 (16.86)	75.74 (16.09)	0.989
Height, mean (SD), m	1.68 (0.08)	1.67 (0.10)	1.67 (0.09)	0.643
Body mass index, mean (SD), kg/m^2^	26.77 (4.87)	27.29 (5.45)	27.04 (5.12)	0.768
Fat mass, %	38.65 (8.18)	39.81 (6.84)	39.26 (7.41)	0.645
Lean mass, %	59.18 (7.66)	58.14 (6.41)	58.63 (6.95)	0.662
Appendicular lean mass, kg/m^2^	7.25 (1.40)	7.08 (1.34)	7.16 (1.35)	0.704

*Note*: Vaccination status at enrolment and medical history findings were assessed by study investigators through a review of medical records and patient interviews conducted during the first study visit. Data are presented as mean (SD) unless otherwise indicated. *p* values were calculated using independent *t*‐tests or Fisher exact tests, as appropriate. No adjustments were made for multiple comparisons. ATC indicates Anatomical Therapeutic Chemical classification. Medication use was categorized according to ATC system groups. “Any medication use” was defined as the presence of ≥ 1 medication across any ATC category. group comparisons were performed using Fisher exact tests where appropriate; V̇O_2_, oxygen uptake; V̇E, minute ventilation; 1RM, one‐repetition maximum.

Cardiopulmonary parameters were comparable between groups, with similar values for peak oxygen uptake (mean [SD], 22.4 [6.0] mL·kg^−1^·min^−1^), ventilatory efficiency (V̇E/V̇CO_2_ slope, 33.7 [4.4]), peak workload, and peak heart rate (all *p* > 0.05). Measures of muscle strength, including chest press, leg press, knee extension, back press, and handgrip strength, did not differ significantly between sequences. Likewise, anthropometric and body composition variables, including body mass index, fat mass percentage, lean mass, and appendicular lean mass, were similar across groups (all *p* > 0.05). The exercise intervention produced the expected cardiorespiratory adaptations: participants in the exercise condition showed significant improvements in V̇O_2_peak, the second ventilatory threshold (VT2), peak workload, and peak heart rate relative to the usual‐care condition, as previously reported in the primary trial publication (Ramírez‐Vélez et al., 2024). Full summaries of pre‐ and post‐intervention CPET values for both treatment periods are provided in Table S2.

### 3.2 Lymphocyte and cytokine treatment effects

**FIGURE 1:**
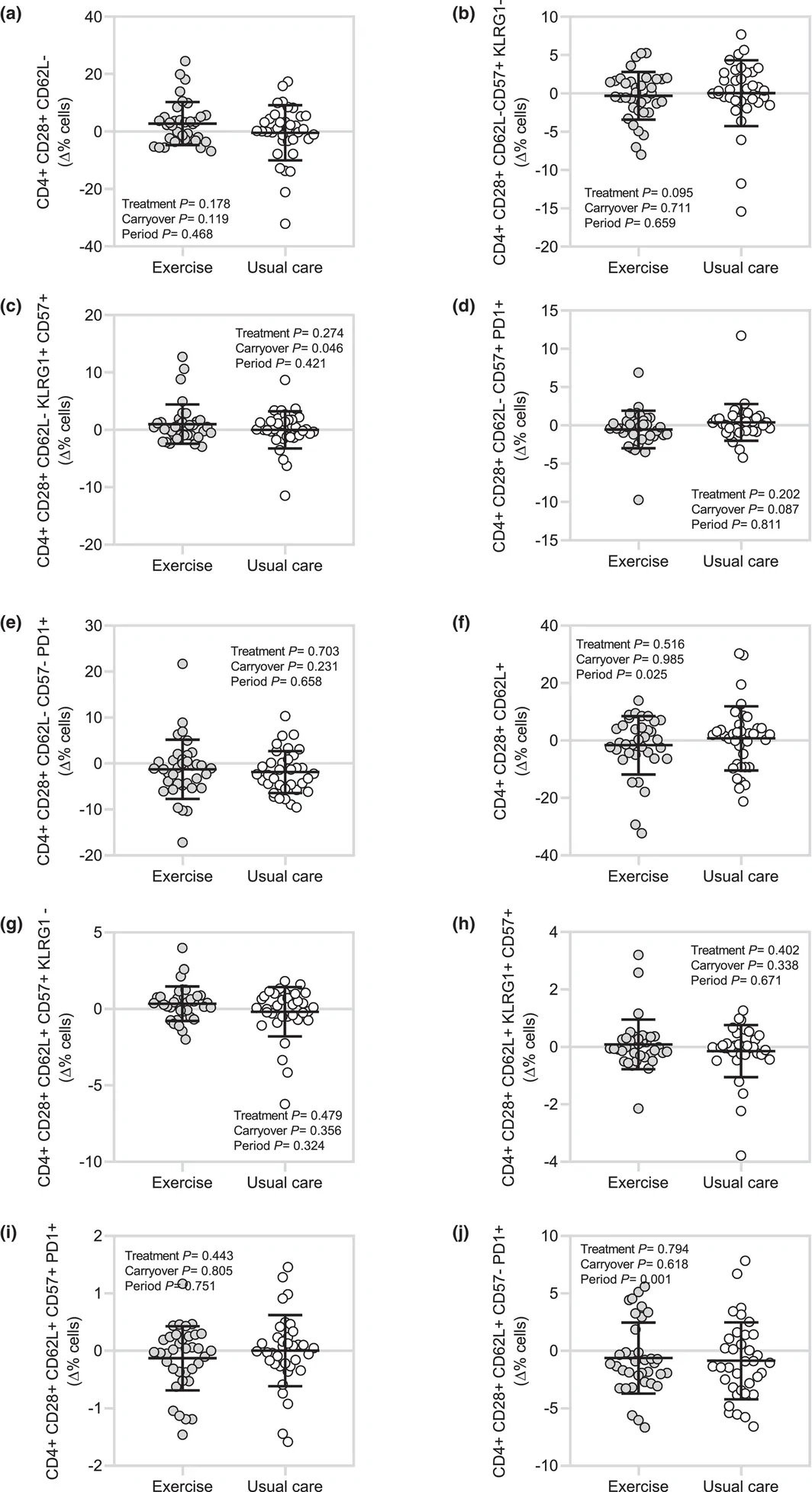
Effects of Exercise on CD4^+^ T‐Cell Subsets in a 2 × 2 Crossover Trial. Changes in circulating CD4^+^ T‐cell subsets after exercise and usual care conditions in a randomized 2 × 2 crossover design. Each panel shows the mean change (Δ%) with corresponding variability (error bars) for specific phenotypic subsets: (a) CD4^+^CD28^+^CD62L^−^, (b) CD4^+^CD28^+^CD62L^−^CD57^+^KLRG1^−^, (c) CD4^+^CD28^+^CD62L^−^KLRG1^+^CD57^+^, (d) CD4^+^CD28^+^CD62L^−^CD57^+^PD1^+^, (e) CD4^+^CD28^+^CD62L^−^CD57^−^PD1^+^, (f) CD4^+^CD28^+^CD62L^+^, (g) CD4^+^CD28^+^CD62L^+^CD57^+^KLRG1^−^, (h) CD4^+^CD28^+^CD62L^+^KLRG1^+^CD57^+^, (i) CD4^+^CD28^+^CD62L^+^CD57^+^PD1^+^, and (j) CD4^+^CD28^+^CD62L^+^CD57^−^PD1^+^. Treatment‐effect *p* values correspond to the pre‐specified one‐sample z test of within‐participant paired treatment contrasts. *p* values for period and carryover effects were obtained from linear mixed‐effects models.

**FIGURE 2:**
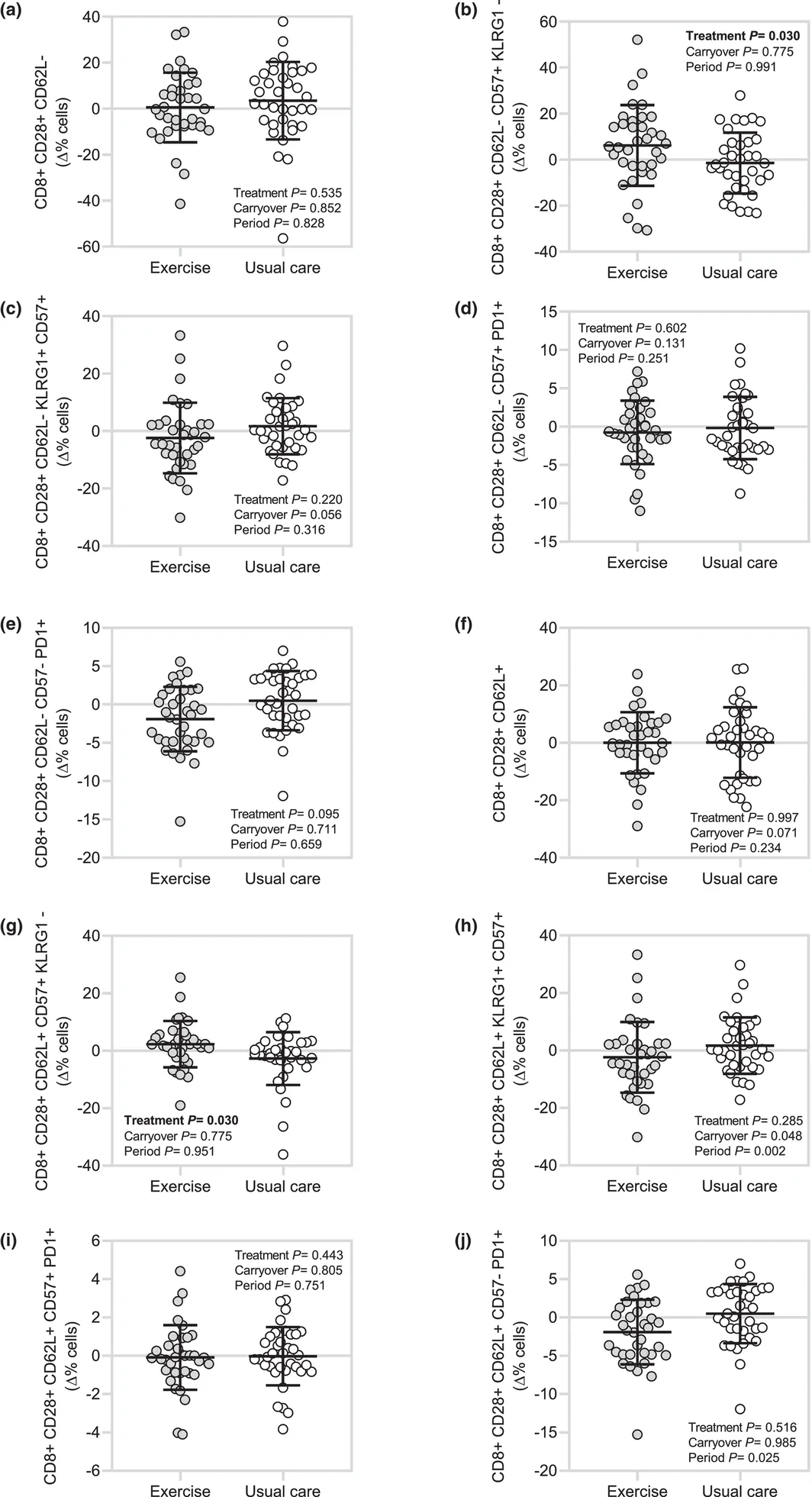
Effects of Exercise on CD8^+^ T‐Cell Subsets in a 2 × 2 Crossover Trial. Changes in circulating CD8^+^ T‐cell subsets under exercise and usual care conditions. Panels represent mean changes (Δ%) across phenotypes: (a) CD8^+^CD28^+^CD62L^−^, (b) CD8^+^CD28^+^CD62L^−^CD57^+^KLRG1^−^, (c) CD8^+^CD28^+^CD62L^−^KLRG1^+^CD57^+^, (d) CD8^+^CD28^+^CD62L^−^CD57^+^PD1^+^, (e) CD8^+^CD28^+^CD62L^−^CD57^−^PD1^+^, (f) CD8^+^CD28^+^CD62L^+^, (g) CD8^+^CD28^+^CD62L^+^CD57^+^KLRG1^−^, (h) CD8^+^CD28^+^CD62L^+^KLRG1^+^CD57^+^, (i) CD8^+^CD28^+^CD62L^+^CD57^+^PD1^+^, and (j) CD8^+^CD28^+^CD62L^+^CD57^−^PD1^+^. Treatment‐effect *p* values correspond to the pre‐specified one‐sample z test of within‐participant paired treatment contrasts. *p* values for period and carryover effects were obtained from linear mixed‐effects models.

**FIGURE 3:**
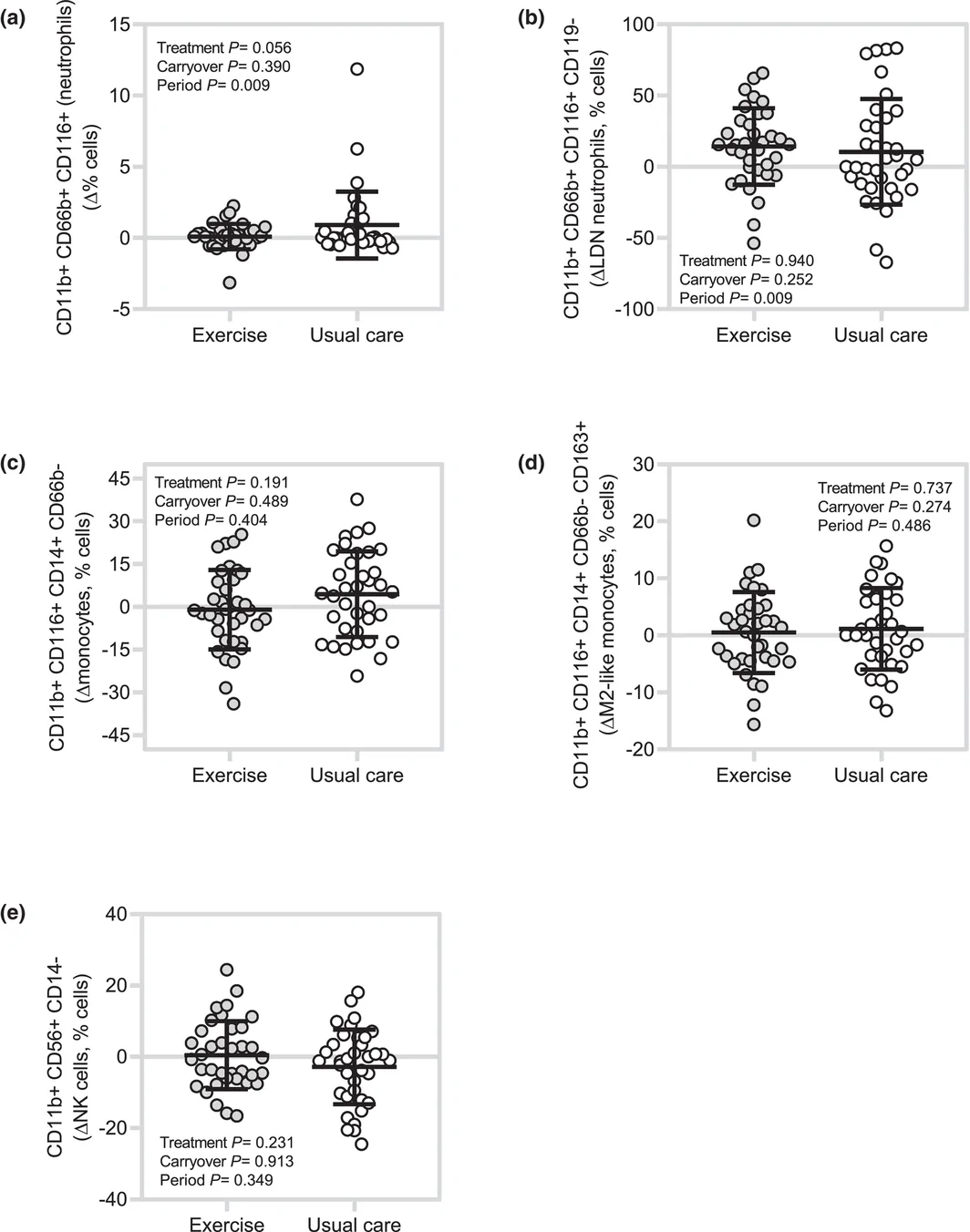
Effects of Exercise on Innate Immune Cell Populations. Changes in innate immune cell populations following exercise and usual care conditions. Panels include (a) neutrophils (CD11b^+^CD66b^+^CD116^+^), (b) low‐density neutrophils (LDN; CD11b^+^CD66b^+^CD116^+^CD119^−^), (c) monocytes, (d) M2‐like monocytes (CD11b^+^CD116^+^CD14^+^CD66b^−^CD163^+^), and (e) natural killer (NK) cells. Treatment‐effect *p* values correspond to the pre‐specified one‐sample z test of within‐participant paired treatment contrasts. *p* values for period and carryover effects were obtained from linear mixed‐effects models.

**FIGURE 4:**
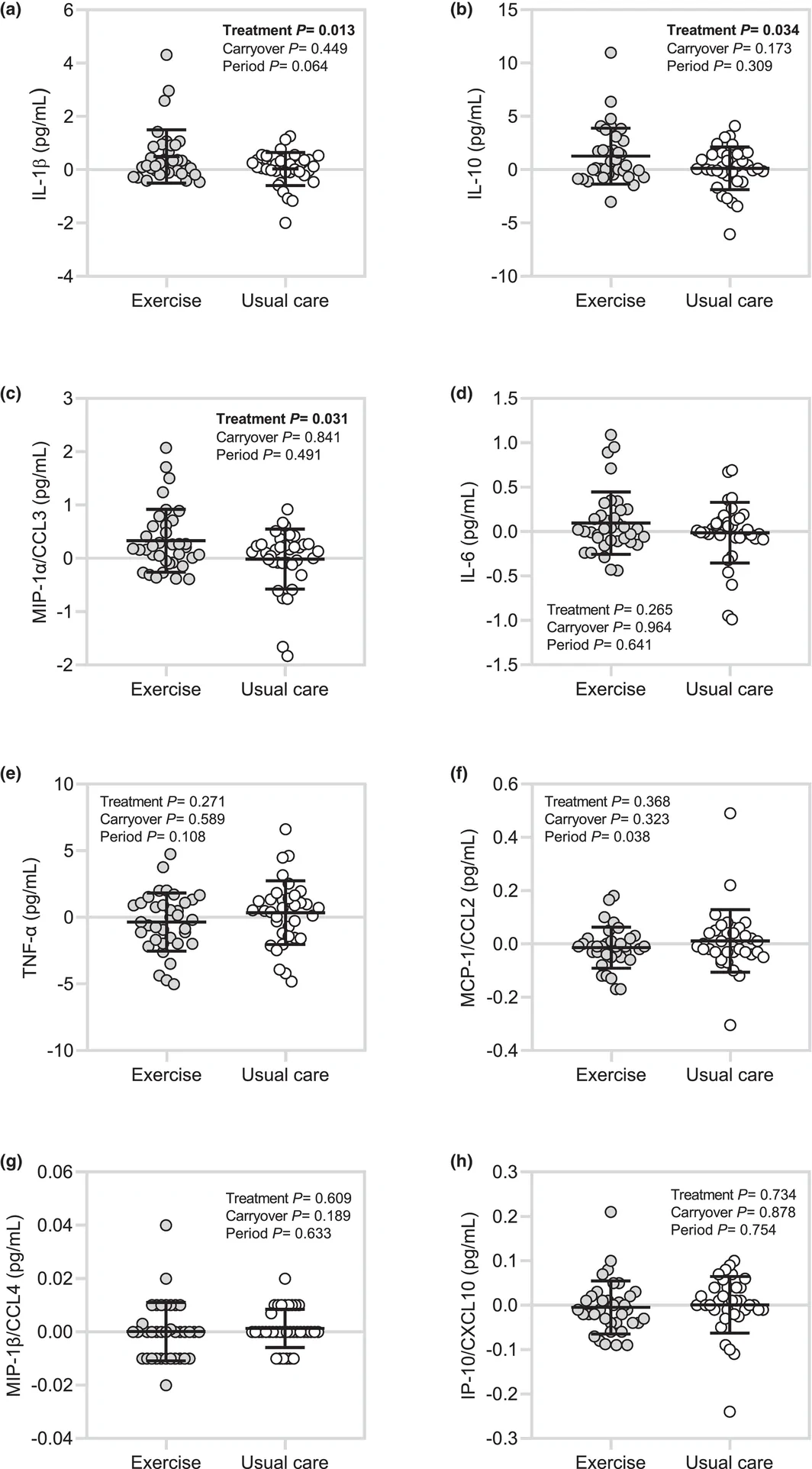
Effects of Exercise on Circulating Cytokines. Changes in circulating cytokine concentrations (pg/mL) under exercise and usual care conditions. Panels show (a) IL‐1β, (b) IL‐10, (c) MIP‐1α/CCL3, (d) IL‐6, (e) TNF‐α, (f) MCP‐1/CCL2, (g) MIP‐1β/CCL4, and (h) IP‐10/CXCL10. Treatment‐effect *p* values correspond to the pre‐specified one‐sample z test of within‐participant paired treatment contrasts. *p* values for period and carryover effects were obtained from linear mixed‐effects models.

Changes in adaptive immune cell subsets, innate immune populations, and circulating cytokines according to treatment condition are shown in Figures 1, 2, 3, 4 and Tables S3 and S4. Treatment effects were heterogeneous across outcomes, with no evidence of consistent between‐condition differences. Of the 29 outcomes in the lymphocyte and cytokine panels, five showed nominal signals before correction (raw *p* < 0.05): CD8^+^CD28^+^CD62L^−^CD57^−^PD1^+^ (−2.40%‐points; *p* = 0.023, Figure 2e), CD8^+^CD28^+^CD62L^+^CD57^+^KLRG1^−^ (+5.03%‐points; *p* = 0.030, Figure 2g), IL‐1β (+0.47 pg/mL; *p* = 0.013, Figure 4a), IL‐10 (+1.14 pg/mL; *p* = 0.034, Figure 4b), and MIP‐1α (+0.34 pg/mL; *p* = 0.031, Figure 4c). However, none of which survived BH adjustment (BH *P* range 0.135–0.149). Nominal evidence of carryover was observed in 4 of 21 CD8⁺ outcomes (19%) before adjustment for multiple testing; however, none remained significant after BH correction. A period effect was identified for CD4⁺CD28⁻CD62L⁺CD57⁻PD‐1⁺, CD8⁺CD28⁻CD62L⁺KLRG1⁺CD57⁺, CD8⁺CD28⁻CD62L⁺CD57⁻PD‐1⁺, and MCP‐1 (CCL2); this did not survive BH correction.

Changes in innate immune cell populations are shown in Figure 3. No outcome in the innate immune panel reached statistical significance after BH correction for the primary treatment effect. The largest nominal treatment signal was for neutrophils (−0.81 percentage points; 95% CI −1.63 to +0.02; *z* = −1.91; raw *p* = 0.056; BH *p* = 0.34). A significant period effect, surviving BH correction, was detected for neutrophils and low‐density neutrophils (BH *p* = 0.029 for both), indicating that values in the second study period were systematically lower than in the first, independent of treatment assignment. No differential carryover effect was detected for any innate immune outcome after BH correction.

### 3.3 Sensitivity analyses and Bayesian analysis of focal outcomes

LMEM estimates were consistent with the primary analysis across five lymphocyte and cytokine focal outcomes (*p* < 0.05). Standard error ratios between the LMEM and change‐score approaches were close to unity for most outcomes (with ratios ranging from 0.90 to 1.01), indicating minimal variance inflation from period or sequence effects. Bayesian crossover models showed adequate convergence, and directional posterior probabilities ranged from 95.4% to 98.9%, indicating a consistent tendency in the direction of the observed treatment contrasts. BF_10_ values ranged from 0.065 to 1.54; four of the five outcomes yielded BF_10_ < 1.0, indicating that the data favor the null hypothesis over the alternative. For IL‐1β and MIP‐1α, the corresponding BF₀₁ values were approximately 8.3 and 15.4, respectively (equivalent to BF₁₀ values of 0.12 and 0.065), representing moderate‐to‐strong evidence in favor of the null hypothesis according to Jeffreys' classification. The single outcome with BF_10_ >1.0 (CD8⁺CD28⁺CD62L⁻CD57⁻PD‐1⁺) yielded BF_10_ = 1.54, representing anecdotal and non‐decisive evidence for an effect. The posterior median for IL‐1β was +0.37 pg/mL (95% CrI −0.01 to +0.78; *p* ≥ 97.2%; BF_10_ = 0.12); for MIP‐1α, +0.26 pg/mL (95% CrI −0.04 to +0.58; *p* ≥ 95.4%; BF_10_ = 0.065). Full posterior summaries for all five focal outcomes are provided in Table S5, whereas posterior summaries for the six innate immune panel outcomes are provided in Table S6. The median degrees‐of‐freedom parameter ν ranged from 10 to 21 across outcomes, indicating modest departures from normality. High directional probability combined with BF_10_ < 1.0 indicates that the posterior distributions are directionally skewed but centred near zero; this pattern does not support inference of clinically meaningful immunomodulatory effects at the concentrations and timepoints assessed. For the five innate immune panel outcomes (Table S6), Bayesian models yielded directional probabilities between 62% and 88% and BF₁₀ values ranging from 0.054 to 0.78, providing anecdotal to moderate evidence in favor of no treatment effect. Posterior medians for monocytes and NK cells were −4.4 percentage points (95% CrI −11.8 to +2.8) and +2.5 percentage points (95% CrI −2.6 to +7.6), respectively; wide credible intervals reflect substantial within‐subject variability in these populations. For all outcomes, posterior estimates were directionally consistent with the frequentist results, but the 95% credible intervals crossed the null value. Bayes factors provided moderate to strong support for the null hypothesis for IL‐1β, IL‐10, and MIP‐1α (BF_10_ = 0.06–0.28), anecdotal support for the alternative hypothesis for CD8 effector memory PD‐1^+^ T cells (BF_10_ = 1.54), and inconclusive evidence for CD8 central memory KLRG1^−^ T cells (BF_10_ = 0.95). All models demonstrated satisfactory convergence (all *R̂* ≤ 1.003).

Exploratory post hoc analyses stratified by sex (Table S7) identified several nominally significant treatment‐associated differences among women, including IL‐1β and selected CD8^+^ memory T‐cell subsets, whereas only one CD8^+^ central memory phenotype reached nominal significance among men. No consistent sex‐specific pattern was observed across cytokines or lymphocyte phenotypes. Given the limited subgroup sample sizes, absence of adjustment for multiple comparisons, and exploratory nature of these analyses, these findings should be interpreted cautiously and regarded as hypothesis‐generating.

## 4 DISCUSSION

Overall, supervised exercise was not associated with statistically significant immunological changes after correction for multiple comparisons. Before adjustment, nominal changes were observed for IL‐1β, IL‐10, and MIP‐1α; however, these findings were attenuated after controlling for multiple testing and should therefore be interpreted as exploratory. Taken together, these findings suggest that the immunomodulatory effects of exercise in this setting are modest, phenotype‐specific, and sensitive to the temporal structure inherent to crossover designs.

These null results are consistent with, and extend, the available literature on exercise immunology in post‐COVID populations. In a trial of COVID‐19 survivors, 12 weeks of supervised training reduced MCP‐1 and interferon‐γ and increased IL‐10 (Filgueira et al., 2023), whereas a ten‐week aerobic training program in long COVID produced no significant change in cytokines or leukocyte subsets despite improvements in cardiorespiratory fitness (Abbasi et al., 2024). The discrepancy across these studies most plausibly reflects differences in intervention duration, patient selection, and training intensity, as well as the heterogeneous immunological trajectories that characterize long COVID as a syndrome. The present study adds a dual‐compartment, pre‐specified assessment within a randomized crossover design—a methodological feature absent from prior immunological substudies—and reaches the same essential conclusion as the negative trial by (Abbasi et al., 2024) brief supervised exercise does not substantially remodel the circulating immune profile in this population.

Contemporary exercise immunology characterizes the immune response to physical training as a process of transient lymphocyte redistribution and functional reprogramming, rather than a uniform pro‐ or anti‐inflammatory shift (Campbell & Turner, 2018; Nieman & Wentz, 2019). This framing is relevant in clinical populations with pre‐existing immunological perturbations, where the immunological effects of exercise may differ from those observed in healthy individuals. Against this background, the directional trends observed for IL‐1β, MIP‐1α, and IL‐10—though not statistically significant—are biologically coherent. IL‐1β is a central mediator of innate inflammatory signaling (Dinarello, 2018), and MIP‐1α (CCL3) is required for leukocyte recruitment and trafficking; modest transient increases in these mediators following exercise may reflect early physiological activation rather than pathological immune engagement. The directional trend toward elevated IL‐10 is consistent with concomitant activation of counter‐regulatory, anti‐inflammatory pathways (Dinarello, 2018; Peake et al., 2017; Turner, 2016). These observations are hypothesis‐generating and require confirmation in adequately powered trials; they do not alter the primary null finding.

The absence of widespread alterations in lymphocyte phenotypes is biologically plausible given the intervention duration and intensity. Sustained changes in circulating T‐cell subset composition typically require longer or more intensive training programmes than those employed here (Theall et al., 2021). Catecholamine release during exercise produces transient increases in circulating CD8+ T cells and natural killer cells, but these acute mobilization effects are rapidly reversible and unlikely to produce durable phenotypic remodeling within a six‐week, twice‐weekly protocol (Nieman & Wentz, 2019). The confinement of nominally significant treatment associations and sequence effects to CD8+ populations—with no comparable signals in CD4+ subsets—is consistent with the known biological properties of CD8+ T cells. These cells are primary effectors of immune surveillance and are particularly susceptible to differentiation‐associated remodeling, including the emergence of late‐differentiated and senescent phenotypes characterized by loss of CD28 and acquisition of CD57, KLRG1, and PD‐1 (Akbar & Henson, 2011; Henson & Akbar, 2009; Nikolich‐Žugich, 2018). These phenotypes display features of replicative senescence, reduced proliferative capacity, and altered cytokine secretion, and their accumulation has been associated with immunosenescence and chronic antigenic stimulation (Akbar & Henson, 2011; Nikolich‐Žugich, 2018). The minor CD8+ changes identified in this study are consistent with established mechanisms of T‐cell differentiation and are unlikely to represent treatment‐specific effects, particularly given their failure to survive multiplicity correction.

The sequence effects confined to CD8+ populations require explicit interpretation in the context of the study design. A washout interval of 3–5 days between two 6‐week immunological exposure periods is insufficient to achieve full phenotypic reversal in the CD8+ compartment. Brief physiological stimuli can induce durable transcriptional and metabolic reprogramming in memory and effector CD8+ T cells (Henson & Akbar, 2009; Kaech & Cui, 2012), and the carryover effects identified in 19% of CD8+ outcomes are consistent with residual immunological imprinting across periods rather than true between‐treatment differences. This represents the principal threat to the internal validity of the cellular treatment estimates, and CD8+ subset findings should be interpreted accordingly. Period effects—characterized by modest declining trends across multiple markers, consistent in direction across treatment sequences—are more plausibly attributable to biological variability, regression to the mean, or ongoing immunological remodeling over time than to the exercise intervention. Circulating T‐cell subset composition fluctuates over brief intervals in response to subclinical antigen exposure, circadian variation, and metabolic state (Pearce & Pearce, 2013), and such dynamics are not fully controlled within the current design. These observations provide structural context for the cytokine panel but do not permit inference about exercise‐induced pathway activation (Davis et al., 2017; Mohamed & Alawna, 2021; Peluso et al., 2021; Pulendran & Davis, 2020; Simpson et al., 2020). The broader literature on immune dysregulation in long COVID—including documented alterations in interferon signaling, T‐cell exhaustion, and cytokine profiles (Phetsouphanh et al., 2022; Su et al., 2022)—provides important background but does not change the interpretation of the present null results.

From a clinical standpoint, the central and actionable finding of this study is the absence of immunological disruption following brief supervised exercise in a population in which immune vulnerability has been a stated concern (Kamphorst et al., 2017; Natale et al., 2003; Wherry & Kurachi, 2015). These findings support the absence of detectable adverse immune perturbations following short‐term, moderate‐intensity supervised exercise in adults with long COVID, including those who do not meet criteria for clinically significant PESE (Ramakrishnan et al., 2021). The modest, non‐significant directional trends for IL‐1β and MIP‐1α, and the exploratory CD8+ associations, identify candidate endpoints and compartments for investigation in future trials. Those trials should incorporate a washout period of at least 4–6 weeks between immunological assessment windows, prespecify a single primary immunological endpoint to constrain the multiple‐testing burden, and include functional immune assays and antigen‐specific readouts to enable mechanistic inference beyond phenotypic characterization (Ezzatvar et al., 2022). The finding that significant functional improvements in long COVID—including gains in V̇O_2_peak, muscle strength, and patient‐reported outcomes documented in the primary EXER‐COVID analysis (Ramírez‐Vélez et al., 2024)—are not accompanied by detectable changes in circulating immune markers suggests that the mechanisms underlying functional benefit in this population do not operate principally through large‐scale immunological remodeling at the concentrations and timepoints assessed here.

An additional consideration in the interpretation of these findings is the clinical and biological heterogeneity of the study population. Individuals with long COVID represent a spectrum of phenotypes that likely reflect distinct underlying mechanisms, including persistent viral reservoirs, immune dysregulation, autonomic dysfunction, and metabolic impairment (Nalbandian et al., 2021; Peluso et al., 2021; Phetsouphanh et al., 2022; Su et al., 2022). This heterogeneity may attenuate detectable group‐level effects and contribute to variability in immunological responses to exercise, thereby reducing the ability to identify consistent treatment‐related changes across outcomes. In this context, the absence of robust signals after multiplicity correction should not be interpreted as evidence of uniform biological inactivity but rather as a reflection of population‐level variability. Furthermore, the biological basis of long COVID remains incompletely defined, and it is plausible that distinct mechanistic subtypes exert differential effects on immune profiles, particularly across cytokine signaling pathways and T‐cell phenotypes (Nalbandian et al., 2021; Phetsouphanh et al., 2022). Such heterogeneity complicates attribution of observed immunological patterns to the intervention and underscores the need for stratified analyses and deep phenotyping strategies in future trials.

### 4.1 Limitations

The first design limitation of this study is the brief washout interval (3–5 days) separating two 6‐week immunological exposure periods. For cytokine endpoints, this duration may be adequate; for cellular immunophenotypes, particularly memory and senescence‐associated CD8+ T‐cell subsets, it is likely insufficient. The sequence (carryover) effects identified in CD8+ populations are consistent with this concern and indicate that treatment estimates for cellular markers may reflect incomplete phenotypic reversal rather than true between‐treatment differences. This limitation cannot be resolved post hoc and constrains causal inference for the cellular compartment. Second, the modest sample size (*n* = 35–36) constrained statistical power to detect small‐to‐moderate treatment effects across a 29‐outcome panel; the FDR correction required to manage this multiplicity burden further reduced sensitivity for individual markers, and the possibility of type II error cannot be excluded. Third, functional pathway annotations were derived from external reference databases and were not experimentally validated in the study cohort. Fourth, high‐resolution temporal cytokine profiling was not performed, precluding characterization of the kinetics of exercise‐induced immune responses across the training period. Fourth, the observational association between exercise and immunological change within a secondary substudy does not permit causal mechanistic inference. Additionally, the cohort was predominantly female (63.9%) with a mean age of 47.7 years (SD 9.7), placing a substantial proportion near or at the menopausal transition. Estrogen withdrawal is associated with altered immune function and blunted exercise‐induced immunomodulation has been reported in post‐menopausal compared with pre‐menopausal women; this composition may have attenuated detectable group‐level immunological responses and represents an important constraint on generalization. Menopausal status was not systematically assessed at enrolment and cannot be formally evaluated post hoc. Post‐hoc exploratory sex‐disaggregated analyses (women *n* = 23, men *n* = 13; Table S7) indicated that the nominally significant cytokine (IL‐1β) and CD8^+^ lymphocyte treatment signals were concentrated predominantly in women, consistent with the known influence of sex hormones on exercise‐induced immunomodulation; however, stratum sizes are insufficient for confirmatory inference and these observations are hypothesis‐generating only.

## 5 CONCLUSIONS

In this randomized crossover trial, short‐term supervised exercise was not associated with significant changes in circulating cytokines or lymphocyte immunophenotypes after multiplicity correction. This null finding supports the immunological safety of twice‐weekly moderate‐intensity supervised exercise in adults with long COVID who do not meet criteria for clinically significant post‐exertional symptom exacerbation. Because participants were systematically screened for worsening post‐exertional symptoms before each session and excluded if clinically significant PESE was present, this safety inference is restricted to the PESE‐negative subgroup. The immunological effects of exercise in PESE‐positive individuals—for whom immune vulnerability from exercise represents the greatest clinical concern—remain uncharacterised and require dedicated study. Cellular immunophenotype estimates, particularly those involving CD8^+^ T‐cell subsets, should be interpreted cautiously. IL‐1β and MIP‐1α (mean differences +0.47 pg/mL and +0.34 pg/mL, respectively) showed the largest exercise‐associated effect sizes in the cytokine panel but did not survive FDR adjustment; these are candidate endpoints for prospectively powered trials. Future studies should prespecify a single primary immunological endpoint, apply a washout interval of at least 4–6 weeks between immunological exposure periods, and incorporate functional immune assays capable of antigen‐specific readouts.

## AUTHOR CONTRIBUTIONS

**Nora García‐Alonso:** Formal analysis; investigation; methodology; resources; visualization. **Mikel Izquierdo:** Conceptualization; data curation; methodology; project administration; validation. **Hugo Arasanz:** Data curation; funding acquisition; methodology; visualization. **Robinson Ramírez‐Vélez:** Conceptualization; data curation; formal analysis; investigation; methodology; supervision; validation; visualization.

## FUNDING INFORMATION

The EXER‐COVID Crossover Study was supported by «Proyectos de I + D + i» de los Programas Estatales de Generación de Conocimiento y Fortalecimiento Científico y Tecnológico del Sistema de I + D + i Orientada a los Retos de la Sociedad, en el marco del Plan Estatal de Investigación Científica y Técnica y de Innovación 2017–2020 (PID2020‐113098RB‐I00).

## CONFLICT OF INTEREST STATEMENT

The authors declare no conflict of interest. No generative AI tool was used to produce, analyze, or interpret experimental data, perform statistical analyses, or create figures. All AI‐assisted text was reviewed, edited, and approved by the authors. The authors take full responsibility for the accuracy, integrity, and originality of the manuscript content.

## ETHICS STATEMENT

The study was conducted in accordance with the Declaration of Helsinki, and the protocol was approved by the Research Ethics Board of Hospital Universitario de Navarra (reference: PI_2020/140), and all participants provided written informed consent before enrolment.

## Supporting information


**Table S1.** Descriptive Statistics by Treatment and Timepoint.
**Table S2.** Cardiopulmonary exercise testing (CPET) outcomes by treatment period.
**Table S3.** Effects of Exercise Intervention on Immune Outcomes.
**Table S4.** Effects of Exercise Intervention on Inflammatory Outcomes (Cytokines).
**Table S5.** Sensitivity Analysis by Linear Mixed‐Effects Models and ANCOVA for Treatment Effects for 2 × 2 Crossover Trial.
**Table S6.** Bayesian Crossover Analysis of Bayesian Crossover Analysis on Focal Outcomes with Nominal Signal.
**Table S7.** Post‐hoc exploratory sex‐disaggregated treatment effects (exercise vs. usual care).

## Data Availability

De‐identified data that support the findings of this study are available from the corresponding author upon reasonable request, subject to institutional ethics and privacy requirements.
