# Supplementary material for: Immunological effects of supervised exercise in long COVID in adults: A secondary analysis of a randomized crossover trial
Source: Physiol Rep. 2026 Aug 28;14(17):e71031. doi: 10.14814/phy2.71031 (PMC13525194; doi:10.14814/phy2.71031)
Supplement: Supplementary file 1 [file PHY2-14-e71031-s001.pdf]

Supplementary files

Immunological Effects of Supervised Exercise in Long COVID in Adults: A Secondary Analysis of a Randomised Crossover Trial

Nora García-Alonso, Ph.D.[1,2], Mikel Izquierdo, Ph.D.[1,2], Hugo Arasanz, Ph.D[3]., Robinson Ramírez-Vélez, Ph.D.[1,2]

1. Navarrabiomed, Hospital Universitario de Navarra (HUN), Universidad Pública de Navarra (UPNA), Instituto de Investigación Sanitaria de Navarra (IdiSNA), Pamplona, España.

2. CIBER of Frailty and Healthy Aging (CIBERFES), Instituto de Salud Carlos III, Madrid, Spain.

3. Medical Oncology Department, Hospital Universitario de Navarra. Oncobiona Group, Navarrabiomed. IdiSNA; Oncoimmunology Group.

***Corresponding author:** Robinson Ramirez-Velez, PhD, E-mail address: robin640@hotmail.com // robinson.ramirez@unavarra.es

Supplementary Methods Analysis: All analyses follow the pre-specified plan for a 2×2 AB/BA crossover design. Within-subject change scores were computed as the difference between end-of-period and start-of-period values for each treatment period. Missing data were handled using multiple imputation by chained equations (MICE) under a missing-at-random assumption. Imputation models included treatment sequence, period, baseline (T1), pre-period values, and all outcomes included in the analysis. A total of 20 imputed datasets were generated, and parameter estimates were combined using Rubin's rules. Results from imputed analyses were consistent with complete-case analyses. Clinical variables were prespecified and analyzed as categorical or continuous variables, as appropriate. Descriptive statistics for lymphocyte subsets, cytokines, and innate immune markers, stratified by treatment group and time point, are provided in Supplementary Table S1.

The primary treatment contrast was the mean difference in change scores between the exercise and standard-care periods, estimated via a one-sample z-test on the paired contrasts (exercise change minus standard-care change). Ninety-five percent confidence intervals were calculated from the empirical standard error of the contrasts. Differential carryover was assessed by a Welch two-sample t-test comparing period-sum scores between sequences (Grizzle test); a period effect was assessed analogously using period-difference scores. Multiple comparisons across all outcomes (lymphocyte, cytokine and innate panel) were controlled using the Benjamini-Hochberg (BH) procedure separately within each panel, with a false discovery rate threshold of 5%.

As sensitivity analyses, treatment effects were re-estimated using a linear mixed-effects model (LMEM) that decomposed within-subject variance into treatment and period components, with standard errors derived from residual variance after removing both effects ($df = n - 2$). Treatment effect estimates were additionally adjusted for baseline values at the first period using analysis of covariance (ANCOVA: post-value ~ treatment + period + baseline T1). For five outcomes with nominal but non-FDR-significant treatment signals (IL-1β, MIP-1α, IL-10, CD8 effector-memory PD-1+ [CD8, EM, PD1+], CD8 central-memory KLRG1− [CD8, CM, KLRG1−]) we estimated Bayesian crossover models using a Student-t likelihood for robustness to outliers: $d_i \sim t_\nu(\mu, \sigma)$, where d_i is the i-th within-subject contrast. Weakly informative priors were assigned as $\mu \sim N(0, 10^2)$, $\sigma \sim$ Half-Normal(10), and $\nu \sim$ Gamma(2, 0.1) + 2. Posterior sampling used adaptive Metropolis-Hastings MCMC (15 000 warmup, 50 000 retained draws per chain; 2 chains). Convergence was assessed by split-chain R-hat; all R-hat values were below 1.01. Evidence for treatment effects was quantified by the Bayes factor (BF_{10}) estimated via the Savage-Dickey density ratio at $\mu = 0$ using Gaussian kernel density estimation (Silverman bandwidth). Directional posterior probability P(direction) and the probability of exceeding a minimum clinically important difference (δ_{min} defined as 10% of the contrast standard deviation) were also reported. All analyses were performed in R (R Foundation for Statistical Computing), using the lme4 and mice packages for mixed-effects modeling and multiple imputation, respectively.

Supplementary Table S1. Descriptive Statistics by Treatment and Timepoint

Outcome	T1 Baseline (all, n=36)	T2 — End of Period 1		T3 — End of Period 2	
		Exercise (A)	Usual care (B)	Exercise (A)	Usual care (B)
**Lymphocyte subpopulations (%)**					
CD4+ CD28− CD62L− (%)	20.45 ± 7.78	22.59 ± 9.35	18.92 ± 6.17	21.32 ± 10.52	24.43 ± 6.27
CD4+ CD28− CD62L− CD57− KLRG1− (%)	6.49 ± 4.00	5.80 ± 3.70	5.32 ± 2.46	5.63 ± 2.81	6.85 ± 3.64
CD4+ CD28− CD62L− CD57− PD1+ (%)	11.03 ± 4.96	9.25 ± 2.56	8.93 ± 2.89	8.55 ± 5.93	7.16 ± 3.09
CD4+ CD28− CD62L− CD57+ PD1+ (%)	2.26 ± 1.40	1.72 ± 1.24	2.49 ± 2.80	2.28 ± 1.99	1.92 ± 1.19
CD4+ CD28− CD62L− KLRG1+ CD57+ (%)	2.71 ± 2.40	3.41 ± 2.69	3.68 ± 2.78	4.88 ± 5.97	2.38 ± 2.05
CD4+ CD28− CD62L+ (%)	70.49 ± 10.97	71.98 ± 12.06	73.74 ± 9.65	70.37 ± 13.12	68.80 ± 9.92
CD4+ CD28− CD62L+ CD57− PD1+ (%)	5.44 ± 2.81	6.18 ± 1.70	5.99 ± 2.49	3.90 ± 1.81	4.00 ± 2.13
CD4+ CD28− CD62L+ CD57+ KLRG1− (%)	3.38 ± 11.69	1.77 ± 1.21	1.18 ± 0.61	1.51 ± 0.98	1.69 ± 0.95
CD4+ CD28− CD62L+ CD57+ PD1+ (%)	0.59 ± 0.57	0.62 ± 0.45	0.51 ± 0.41	0.37 ± 0.23	0.58 ± 0.38
CD4+ CD28− CD62L+ KLRG1+ CD57+ (%)	0.54 ± 0.64	0.67 ± 0.93	0.48 ± 0.49	0.56 ± 0.64	0.41 ± 0.42
CD8+ CD28− CD62L− (%)	44.48 ± 15.55	51.77 ± 14.83	40.88 ± 12.78	42.12 ± 20.55	55.54 ± 18.28
CD8+ CD28− CD62L− CD57− PD1+ (%)	7.61 ± 5.46	5.97 ± 3.02	8.77 ± 4.12	6.44 ± 2.83	5.94 ± 2.38
CD8+ CD28− CD62L− CD57+ KLRG1− (%)	40.57 ± 18.43	49.20 ± 17.37	35.01 ± 18.81	42.56 ± 15.99	48.34 ± 15.44
CD8+ CD28− CD62L− CD57+ PD1+ (%)	5.83 ± 3.42	6.02 ± 2.87	6.12 ± 2.77	5.18 ± 3.27	4.60 ± 2.05
CD8+ CD28− CD62L− KLRG1+ CD57+ (%)	9.82 ± 7.17	9.71 ± 6.14	12.60 ± 9.65	10.11 ± 12.30	8.01 ± 7.44
CD8+ CD28− CD62L+ (%)	43.74 ± 14.75	40.14 ± 11.46	50.73 ± 14.78	50.57 ± 16.54	36.30 ± 13.25
CD8+ CD28− CD62L+ CD57− PD1+ (%)	6.00 ± 2.88	10.49 ± 10.12	11.93 ± 15.58	5.30 ± 3.07	6.45 ± 2.27
CD8+ CD28− CD62L+ CD57+ KLRG1− (%)	12.64 ± 10.95	17.82 ± 8.67	7.20 ± 5.51	9.34 ± 9.14	15.43 ± 7.13
CD8+ CD28− CD62L+ CD57+ PD1+ (%)	2.01 ± 1.53	2.64 ± 1.75	1.87 ± 1.17	1.58 ± 1.50	2.24 ± 0.96
CD8+ CD28− CD62L+ KLRG1+ CD57+ (%)	5.65 ± 3.80	7.83 ± 3.45	5.33 ± 5.25	3.94 ± 4.11	3.80 ± 2.88
**Innate immune cells (%)**					
Monocytes (CD14+CD66b−) (%)	28.30 ± 10.85	25.82 ± 11.78	30.84 ± 14.53	32.34 ± 13.11	31.22 ± 12.44
M2-like monocytes (CD163+) (%)	74.46 ± 6.62	76.04 ± 6.69	75.98 ± 7.10	76.52 ± 3.99	75.69 ± 6.69
NK Cells (CD56+CD14−) (%)	35.11 ± 13.27	34.98 ± 7.82	30.40 ± 9.74	31.93 ± 10.23	33.61 ± 10.67
Neutrophils (CD66b+) (%)	0.85 ± 0.67	0.63 ± 0.22	0.97 ± 0.99	1.42 ± 1.34	2.33 ± 3.11
LDN neutrophils ($CD11b^{+}CD66b^{+}CD116^{+}CD119^{-}$)	27.10 ± 16.91	27.09 ± 15.43	24.08 ± 19.13	43.91 ± 25.25	54.08 ± 29.87
**Cytokines**					
IL-10 (pg/mL)	4.25 ± 2.12	3.84 ± 3.23	5.34 ± 2.01	7.21 ± 4.02	3.85 ± 2.28
IL-1β (pg/mL)	1.18 ± 0.49	1.33 ± 0.60	1.18 ± 0.55	1.91 ± 1.44	1.46 ± 0.81
IP-10 (pg/mL)	0.18 ± 0.07	0.22 ± 0.08	0.15 ± 0.05	0.14 ± 0.06	0.22 ± 0.07
IL-6 (pg/mL)	0.62 ± 0.33	0.76 ± 0.40	0.53 ± 0.12	0.65 ± 0.40	0.77 ± 0.32
MCP-1 (pg/mL)	0.31 ± 0.17	0.34 ± 0.22	0.22 ± 0.07	0.23 ± 0.06	0.39 ± 0.33
MIP-1α (pg/mL)	1.50 ± 0.42	1.64 ± 0.58	1.55 ± 0.32	1.93 ± 0.70	1.69 ± 0.51
MIP-1β (pg/mL)	0.03 ± 0.01	0.03 ± 0.01	0.03 ± 0.01	0.03 ± 0.02	0.03 ± 0.01
TNF-α (pg/mL)	7.83 ± 2.55	7.29 ± 2.33	7.39 ± 2.28	7.40 ± 2.29	8.29 ± 2.05

Legend: Values are Mean ± SD. T1 = pre-intervention baseline; T2 = end of period 1 (AB sequence received Exercise, BA sequence received Standard Care); T3 = end of period 2 (AB received Standard Care, BA received Exercise). Exercise (A): supervised structured exercise programme. Standard Care (B): usual medical care.

Supplementary Table S2. Cardiopulmonary exercise testing (CPET) outcomes by treatment period

Outcome			Exercise Period			Usual-Care Period			Between-Condition Contrast				
**Outcome**	**Unit**	**n**	**Pre (mean±SD)**	**Post (mean±SD)**	**Δ**	**Pre (mean±SD)**	**Post (mean±SD)**	**Δ**	**Δ Ex − Δ UC**	**95% CI**	**t**	**p**	**p_adj (BH)**
VO_2peak	ml·kg⁻¹·min⁻¹	36	22.7 ± 6.2	26.7 ± 6.2	**+4.0**	24.6 ± 6.8	23.7 ± 6.6	−0.9	**+4.82**	[+2.97, +6.68]	5.089	**<0.001**	**<0.001**
VO_2 at VT1	ml·kg⁻¹·min⁻¹	35	9.3 ± 2.2	9.7 ± 2.4	+0.5	9.6 ± 1.9	9.4 ± 2.3	−0.2	+0.66	[−0.64, +1.95]	0.995	0.327	0.327
VO_2 at VT2	ml·kg⁻¹·min⁻¹	35	21.4 ± 5.5	23.4 ± 5.4	**+2.0**	22.6 ± 5.6	21.7 ± 5.4	−0.9	**+2.93**	[+1.14, +4.71]	3.205	**0.003**	**0.005**
Peak Workload	W	35	130.7 ± 46.2	152.1 ± 45.1	**+21.4**	139.3 ± 49.7	137.9 ± 44.3	−1.4	**+22.86**	[+14.73, +30.99]	5.512	**<0.001**	**<0.001**
Peak Heart Rate	bpm	32	156.6 ± 17.1	161.7 ± 17.2	**+5.1**	159.6 ± 18.5	156.8 ± 17.4	−2.8	**+7.84**	[+0.88, +14.80]	2.209	**0.035**	**0.043**

Legend: Δ = within-period change (Post − Pre). Between-condition contrast = Δ Exercise − Δ Usual Care computed per subject and tested against zero using a one-sample t-test. 95% CI = mean ± 1.96 × SE of individual contrasts. p_adj: Benjamini–Hochberg FDR correction at 5%. Gold shading: p_adj < 0.05. VT1/VT2 = first/second ventilatory threshold; UC = usual care; BH = Benjamini–Hochberg. AB group (n=17): exercise Period 1 (T1→T2), usual care Period 2 (T2→T3). BA group (n=19): usual care Period 1 (T1→T2), exercise Period 2 (T2→T3).

Supplementary Table S3. Effects of Exercise Intervention on Immune Outcomes

Outcome	n	Exercise Δ, mean (SD)	Usual Care Δ, mean (SD)	Adjusted Difference (95% CI)	P Value	P Value (BH)
**Selected immune cell subsets**						
CD8^{+}CD28^{+}CD62L^{-}CD57^{-}PD1^{+}	36	−1.90 (4.22)	0.50 (3.86)	−2.40 (−4.47 to −0.33)	0.023	0.149
CD8^{+}CD28^{+}CD62L^{+}CD57^{+}KLRG1^{-}	36	2.30 (8.05)	−2.73 (9.20)	5.03 (0.48 to 9.57)	0.030	0.149
CD8^{+}CD28^{+}CD62L^{-}CD57^{+}KLRG1^{-}	36	6.17 (17.56)	−1.45 (13.28)	7.62 (−1.33 to 16.57)	0.095	0.368
CD4^{+}CD28^{+}CD62L^{-}	36	2.75 (7.46)	−0.42 (9.61)	3.17 (−1.44 to 7.78)	0.178	0.553
CD4^{+}CD28^{+}CD62L^{-}CD57^{+}PD1^{+}	36	−0.54 (2.45)	0.40 (2.40)	−0.94 (−2.39 to 0.51)	0.202	0.553
CD8^{+}CD28^{+}CD62L^{-}KLRG1^{+}CD57^{+}	36	−2.39 (12.31)	1.69 (9.80)	−4.07 (−10.58 to 2.44)	0.220	0.553
CD4^{+}CD28^{+}CD62L^{-}KLRG1^{+}CD57^{+}	36	1.00 (3.43)	−0.01 (3.23)	1.01 (−0.80 to 2.82)	0.275	0.553
CD8^{+}CD28^{+}CD62L^{+}KLRG1^{+}CD57^{+}	36	−0.23 (3.27)	−1.54 (4.69)	1.31 (−1.09 to 3.71)	0.286	0.553
CD4^{+}CD28^{+}CD62L^{+}	36	−2.02 (10.27)	1.16 (11.04)	−3.18 (−9.71 to 3.35)	0.340	0.619
CD4^{+}CD28^{+}CD62L^{+}KLRG1^{+}CD57^{+}	36	0.09 (0.87)	−0.14 (0.90)	0.23 (−0.31 to 0.78)	0.402	0.656
CD4^{+}CD28^{+}CD62L^{+}CD57^{+}PD1^{+}	36	−0.13 (0.56)	0.00 (0.62)	−0.13 (−0.47 to 0.21)	0.443	0.687
CD4^{+}CD28^{+}CD62L^{+}CD57^{+}KLRG1^{-}	36	−1.60 (11.76)	−0.18 (1.62)	−1.42 (−5.36 to 2.52)	0.479	0.708
CD8^{+}CD28^{+}CD62L^{+}CD57^{-}PD1^{+}	36	−1.65 (14.82)	1.50 (14.58)	−3.15 (−12.68 to 6.37)	0.516	0.722
CD8^{+}CD28^{+}CD62L^{-}	36	0.52 (15.12)	3.46 (16.83)	−2.95 (−12.27 to 6.38)	0.536	0.722
CD8^{+}CD28^{+}CD62L^{-}CD57^{+}PD1^{+}	36	−0.76 (4.14)	−0.17 (4.06)	−0.59 (−2.94 to 1.75)	0.621	0.770
CD4^{+}CD28^{+}CD62L^{-}CD57^{-}PD1^{+}	36	−1.26 (6.45)	−1.87 (4.57)	0.61 (−2.54 to 3.76)	0.703	0.825
CD4^{+}CD28^{+}CD62L^{-}CD57^{-}KLRG1^{-}	36	−0.31 (3.11)	0.04 (4.29)	−0.35 (−2.46 to 1.76)	0.745	0.825
CD4^{+}CD28^{+}CD62L^{+}CD57^{-}PD1^{+}	36	−0.63 (3.09)	−0.87 (3.34)	0.23 (−1.53 to 1.99)	0.795	0.850
CD8^{+}CD28^{+}CD62L^{+}CD57^{+}PD1^{+}	36	−0.09 (1.69)	−0.02 (1.52)	−0.07 (−1.00 to 0.87)	0.890	0.919
CD8^{+}CD28^{+}CD62L^{+}	36	0.00 (10.62)	0.09 (12.26)	−0.09 (−6.50 to 6.32)	0.978	0.978
**Innate immune cells**						
Monocytes (CD11b^{+}CD116^{+}CD14^{+}CD66b^{-})	36	−0.96 (13.88)	4.47 (14.97)	−5.42 (−13.55 to 2.71)	0.191	0.347
M2 macrophages (CD163^{+})	36	0.51 (7.10)	1.17 (7.13)	−0.66 (−4.51 to 3.19)	0.737	0.885
NK cells (CD11b^{+}CD56^{+}CD14^{-})	36	0.43 (9.55)	−2.82 (10.47)	3.25 (−2.07 to 8.58)	0.231	0.347
Neutrophils (CD11b^{+}CD66b^{+}CD116^{+})	36	0.10 (0.89)	0.90 (2.35)	−0.81 (−1.63 to 0.02)	0.056	0.337
LDN neutrophils (CD11b^{+}CD66b^{+}CD116^{+}CD119^{-})	36	11.16 (32.28)	10.45 (37.11)	0.71 (−18.75 to 20.16)	0.943	0.943

Legend: Values are presented as mean (SD) within-period changes (Δ) from pre-period measurements (T1 for period 1 and T2 for period 2). The adjusted between-condition difference represents the estimated mean difference (exercise minus usual care) derived from linear mixed-effects models including fixed effects for treatment, period, and sequence, and a random intercept for participant. All analyses accounted for the 2×2 crossover design (AB/BA), with appropriate modeling of within-

subject correlation. P values for treatment correspond to the primary contrast of interest (exercise vs usual care). P values for carryover and period effects were derived from the same mixed-effects models and were provided to assess potential residual and temporal effects inherent to crossover designs. Adjustment for multiple comparisons across outcomes was performed using the Benjamini–Hochberg procedure; adjusted P values (P [BH]) are reported. No outcomes remained statistically significant after correction for multiple comparisons. Negative values indicate lower values under the exercise condition relative to usual care. Estimates should be interpreted with caution owing to variability in several outcomes and the presence of period effects in selected variables.

Supplementary Table S4. Effects of Exercise Intervention on Inflammatory Outcomes (Cytokines)

Outcome (pg/mL)	n	Exercise Δ, mean (SD)	Usual Care Δ, mean (SD)	Adjusted Difference (95% CI)	P Value	P Value (BH)
IL-1β	36	0.50 (1.00)	0.03 (0.62)	0.47 (0.10 to 0.85)	0.013	0.136
MIP-1α	36	0.33 (0.59)	−0.01 (0.56)	0.34 (0.03 to 0.65)	0.031	0.149
IL-10	36	1.26 (2.62)	0.12 (2.00)	1.14 (0.09 to 2.20)	0.034	0.149
IL-6	36	0.10 (0.35)	−0.01 (0.34)	0.11 (−0.08 to 0.30)	0.265	0.553
TNF-α	36	−0.36 (2.19)	0.35 (2.39)	−0.71 (−1.97 to 0.55)	0.271	0.553
MCP-1	35	−0.01 (0.08)	0.01 (0.12)	−0.03 (−0.08 to 0.03)	0.368	0.634
MIP-1β	36	0.00 (0.01)	0.00 (0.01)	−0.00 (−0.01 to 0.00)	0.609	0.770
IP-10	36	−0.00 (0.06)	0.00 (0.06)	−0.01 (−0.04 to 0.03)	0.734	0.825

Legend: Values are presented as mean (SD) within-period changes (Δ) from pre-period measurements (T1 for period 1 and T2 for period 2). The adjusted between-condition difference represents the estimated mean difference (exercise minus usual care) derived from linear mixed-effects models including fixed effects for treatment, period, and sequence, and a random intercept for participant. All analyses accounted for the 2×2 crossover design (AB/BA), with appropriate modeling of within-subject correlation. P values for treatment correspond to the primary contrast of interest (exercise vs usual care). P values for carryover and period effects were derived from the same mixed-effects models and were provided to assess potential residual and temporal effects inherent to crossover designs. Adjustment for multiple comparisons across outcomes was performed using the Benjamini–Hochberg procedure; adjusted P values (P [BH]) are reported. No outcomes remained statistically significant after correction for multiple comparisons. Negative values indicate lower values under the exercise condition relative to usual care. Estimates should be interpreted with caution owing to variability in several outcomes and the presence of period effects in selected variables. Cytokines: Interleukin-1β (IL-1β), interleukin-6 (IL-6), interleukin-10 (IL-10), tumor necrosis factor-α (TNF-α), monocyte chemoattractant protein-1 (MCP-1/CCL2), macrophage inflammatory protein-1α (MIP-1α/CCL3), macrophage inflammatory protein-1β (MIP-1β/CCL4), and interferon-γ–inducible protein-10 (IP-10/CXCL10).

Supplementary Table S5. Sensitivity Analysis by Linear Mixed-Effects Models and ANCOVA for Treatment Effects for 2×2 Crossover Trial

Outcome	n	Change-Score Estimate (SE)	z Statistic	LMEM Estimate (SE)	z Statistic	SE Ratio (LMEM/CS)	ANCOVA Estimate (SE)	p BH Treatment
**Selected immune cell subsets**								
$CD8^+CD28^+CD62L^-CD57^-PD1^+$	36	−2.40 (1.06)	−2.27	1.06 (0.21)	−2.21	1.00	−1.15 (0.69)	0.64
$CD8^+CD28^+CD62L^+CD57^+KLRG1^-$	36	5.03 (2.32)	2.17	2.35 (0.21)	2.13	1.02	2.26 (1.80)	0.66
$CD8^+CD28^+CD62L^-CD57^+KLRG1^-$	36	7.62 (4.56)	1.67	4.62 (0.44)	1.63	1.01	4.20 (2.98)	0.64
$CD4^+CD28^+CD62L^-$	36	3.17 (2.35)	1.35	2.37 (0.55)	1.30	1.01	1.33 (0.74)	0.64
$CD4^+CD28^+CD62L^-CD57^+PD1^+$	36	−0.94 (0.74)	−1.28	0.75 (0.55)	−1.27	1.01	0.28 (1.91)	0.96
$CD8^+CD28^+CD62L^-KLRG1^+CD57^+$	36	−4.07 (3.32)	−1.23	3.32 (0.55)	−1.17	1.00	−0.20 (0.46)	0.92
$CD4^+CD28^+CD62L^-KLRG1^+CD57^+$	36	1.01 (0.92)	1.09	0.93 (0.55)	1.13	1.01	1.12 (0.77)	0.64
$CD8^+CD28^+CD62L^+KLRG1^+CD57^+$	36	1.31 (1.22)	1.07	1.08 (0.53)	1.39	0.88	−0.40 (2.15)	0.96
$CD4^+CD28^+CD62L^+$	36	−3.18 (3.33)	−0.96	3.24 (0.65)	−0.89	0.97	−0.09 (1.90)	0.96
$CD4^+CD28^+CD62L^+KLRG1^+CD57^+$	36	0.23 (0.28)	0.84	0.28 (0.65)	0.85	1.01	0.17 (0.15)	0.68
$CD4^+CD28^+CD62L^+CD57^+PD1^+$	36	−0.13 (0.17)	−0.77	0.18 (0.69)	−0.74	1.01	0.21 (0.23)	0.75
$CD4^+CD28^+CD62L^+CD57^+KLRG1^-$	36	−1.42 (2.01)	−0.71	2.01 (0.69)	−0.77	1.00	−0.05 (0.09)	0.88
$CD8^+CD28^+CD62L^+CD57^-PD1^+$	36	−3.15 (4.86)	−0.65	4.59 (0.78)	−0.56	0.94	−1.26 (3.18)	0.92
$CD8^+CD28^+CD62L^-$	36	−2.95 (4.76)	−0.62	4.82 (0.76)	−0.62	1.01	−0.36 (0.70)	0.89
$CD8^+CD28^+CD62L^-CD57^+PD1^+$	36	−0.59 (1.20)	−0.49	1.19 (0.78)	−0.43	1.00	0.05 (0.49)	0.96
$CD4^+CD28^+CD62L^-CD57^-PD1^+$	36	0.61 (1.61)	0.38	1.63 (0.80)	0.35	1.01	0.24 (0.66)	0.92
$CD4^+CD28^+CD62L^-CD57^-KLRG1^-$	36	−0.35 (1.08)	−0.33	1.06 (0.78)	−0.42	0.98	−1.29 (2.26)	0.88
$CD4^+CD28^+CD62L^+CD57^-PD1^+$	36	0.23 (0.90)	0.26	0.78 (0.78)	0.50	0.86	0.86 (0.91)	0.75
$CD8^+CD28^+CD62L^+CD57^+PD1^+$	36	−0.07 (0.48)	−0.14	0.47 (0.97)	−0.07	0.99	1.84 (2.38)	0.76
$CD8^+CD28^+CD62L^+$	36	−0.09 (3.27)	−0.03	3.25 (0.97)	0.04	0.99	0.06 (0.31)	0.96
**Innate immune cells**								
Monocytes ($CD11b^+CD116^+CD14^+CD66b^-$)	36	−5.42 (4.15)	−1.31	−1.30 (0.36)	−3.48	1.00	−1.95 (2.99)	0.62
M2 macrophages ($CD163^+$)	36	−0.66 (1.97)	−0.34	−0.33 (0.89)	1.40	1.01	0.44 (1.43)	0.76
NK cells ($CD11b^+CD56^+CD14^-$)	36	3.25 (2.72)	1.20	1.19 (0.36)	−2.53	1.00	1.45 (1.88)	0.62
Neutrophils ($CD11b^+CD66b^+CD116^+$)	36	−0.81 (0.42)	−1.91	−2.11 (0.25)	−1.14	0.90	−0.62 (0.41)	0.62
LDN neutrophils ($CD11b^+CD66b^+CD116^+CD119^-$)	36	0.71 (9.93)	0.07	0.08 (0.94)	−24.85	0.92	−3.58 (5.42)	0.62
**Cytokines (pg/mL)**								
IL-1β	36	0.47 (0.19)	2.48	0.18 (0.20)	2.46	0.97	0.30 (0.21)	0.64
IL-10	36	1.14 (0.54)	2.12	0.54 (0.21)	2.07	1.00	0.93 (0.65)	0.64
MIP-1α	36	0.34 (0.16)	2.16	0.16 (0.21)	2.11	1.01	0.16 (0.12)	0.66
TNF-α	36	−0.71 (0.64)	−1.10	0.63 (0.55)	−1.21	0.98	−0.49 (0.46)	0.68
IL-6	36	0.11 (0.10)	1.11	0.10 (0.56)	1.08	1.01	0.05 (0.07)	0.76

MCP-1	35	−0.03 (0.03)	−0.90	0.03 (0.58)	−1.01	0.95	−0.02 (0.02)	0.68
MIP-1β	36	−0.00 (0.00)	−0.51	0.00 (0.78)	−0.48	1.01	0.00 (0.00)	0.96
IP-10	36	−0.01 (0.02)	−0.34	0.02 (0.80)	−0.32	1.01	−0.00 (0.01)	0.96

Legend: Change-score (CS) estimates were calculated as within-period differences (post minus pre). LMEM: SE computed from within-subject residuals after removing treatment + period effects (df = n−2). ANCOVA: Post Value ~ Treatment + Period + Baseline T1 (OLS). SE ratio < 1 = LMEM more efficient than change-score. All P values were adjusted using the Benjamini–Hochberg procedure across all outcomes.

Supplementary Table S6. Bayesian Crossover Analysis of Bayesian Crossover Analysis on Focal Outcomes with Nominal Signal

Outcome	Frequentist Reference			Bayesian Posterior (Student-t likelihood)				Posterior Probabilities						Bayes Factor		MCMC Diagnostics			
	Freq Contrast	Freq SE	p-value (two-tailed)	Post. Mean μ	Post. Median μ	95% CrI Lower	95% CrI Upper	Post. ν (df)	Post. σ median	P(direction) >0 or <0	Interpret.	δ_min	P(|μ|>δ_min)	BF_{10}	BF Interpretation	$\hat{R}$ μ	$\hat{R}$ σ	$\hat{R}$ ν	MCMC n
IL-1β (pg/mL)	0.47	0.19	0.0131	0.38	0.37	-0.01	0.78	15.8	1.06	0.97	>95%	0.1	0.92	0.12	Moderate H0	1.000	1.000	1.000	50000
CD8 EM PD1+ (neg)	-2.4	1.06	0.0231	-2.54	-2.55	-4.66	-0.39	18.8	6.05	0.99	>95%	1	0.92	1.54	Anecdotal H1	1.000	1.000	1.000	50000
CD8 CM KLRG1- (%)	5.03	2.32	0.0302	3.66	3.6	-0.37	7.96	10.2	11.52	0.96	>95%	2	0.79	0.95	Anecdotal H0	1.001	1.001	1.003	50000
MIP-1α (pg/mL)	0.34	0.16	0.0306	0.26	0.26	-0.04	0.58	13	0.85	0.95	>95%	0.1	0.85	0.07	Strong H0	1.000	1.000	1.000	50000
IL-10 (pg/mL)	1.14	0.54	0.0337	0.96	0.95	-0.09	2.04	14.3	2.93	0.97	>95%	0.2	0.92	0.28	Moderate H0	1.000	1.000	1.000	50000

Legend: Priors: $\mu \sim N(0,10^2)$ | $\sigma \sim$ HalfNormal(10) | $\nu \sim$ Gamma(2,0.1)+2 | MH 15k warmup + 50k draws | BF_{10} Savage-Dickey | R-hat convergence diagnostic. P(direction): posterior probability the effect is in the group-level direction | P(|μ|>δ): probability of exceeding 10% of the contrast SD (minimal effect) | BF_{10}: <1/3 = moderate H_0 | <1 = anecdotal H_0 | 1–3 = anecdotal H_1 | >10 = strong H_1

Supplementary Table S7. Post-hoc exploratory sex-disaggregated treatment effects (exercise vs usual care)

Outcome / Phenotype	All (n=34–36) Mean (95% CI)	P	Women (n=22–23) Mean (95% CI)	P	Men (n=11–13) Mean (95% CI)	P
**Cytokine panel (pg/mL)**						
IL-1β	+0.450 (0.058 to 0.841)	**0.024**	+0.496 (0.018 to 0.974)	**0.042**	+0.364 (−0.342 to 1.070)	0.312
IL-6	+0.070 (−0.113 to 0.253)	0.451	+0.111 (−0.141 to 0.363)	0.388	−0.004 (−0.248 to 0.240)	0.973
IL-10	+1.227 (0.118 to 2.337)	**0.030**	+1.156 (−0.301 to 2.614)	0.120	+1.357 (−0.381 to 3.096)	0.126
IP-10/CXCL10	−0.003 (−0.036 to 0.031)	0.878	−0.002 (−0.052 to 0.047)	0.928	−0.003 (−0.038 to 0.031)	0.849
MCP-1/CCL2	−0.875 (−2.494 to 0.744)	0.289	−1.256 (−3.650 to 1.138)	0.304	−0.078 (−0.180 to 0.024)	0.132
MIP-1α/CCL3	+0.291 (−0.025 to 0.606)	0.071	+0.298 (−0.147 to 0.742)	0.190	+0.279 (−0.129 to 0.687)	0.180
MIP-1β/CCL4	−0.001 (−0.006 to 0.004)	0.646	−0.003 (−0.009 to 0.004)	0.399	+0.002 (−0.007 to 0.010)	0.694
TNF-α	−1.041 (−2.248 to 0.166)	0.091	−0.650 (−1.866 to 0.566)	0.295	−1.758 (−4.391 to 0.876)	0.191
**$CD8^+$ lymphocyte**						
$CD8^+$ Effector Memory ($CD28^-MLCD62L^-$)	−2.95 (−12.272 to 6.379)	0.536	−8.09 (−18.969 to 2.791)	0.145	+6.15 (−10.490 to 22.794)	0.469
$CD8^+$ EM $CD57^-PD1^+$	−2.40 (−4.467 to −0.328)	**0.023**	−3.54 (−5.783 to −1.292)	**0.002**	−0.38 (−4.406 to 3.642)	0.852
$CD8^+$ EM $CD57^-KLRG1^-$	+7.62 (−1.326 to 16.565)	0.095	+11.40 (0.160 to 22.649)	**0.047**	+0.92 (−13.699 to 15.544)	0.902
$CD8^+$ EM $CD57^-PD1^+$	−0.59 (−2.938 to 1.754)	0.621	−0.21 (−2.464 to 2.041)	0.854	−1.26 (−6.534 to 4.004)	0.638
$CD8^+$ EM $KLRG1^-CD57^-$	−4.07 (−10.581 to 2.435)	0.220	−4.66 (−13.655 to 4.337)	0.310	−3.04 (−11.926 to 5.856)	0.503
$CD8^+$ Central Memory ($CD28^-MLCD62L^-$)	−0.09 (−6.499 to 6.316)	0.978	+0.96 (−5.703 to 7.621)	0.778	−1.95 (−15.585 to 11.685)	0.779
$CD8^+$ CM $CD57^-PD1^+$	−3.15 (−12.679 to 6.370)	0.516	+1.24 (−6.219 to 8.695)	0.745	−10.93 (−33.775 to 11.922)	0.349
$CD8^+$ CM $CD57^-KLRG1^-$	+5.03 (0.481 to 9.571)	**0.030**	+4.29 (0.094 to 8.484)	**0.045**	+6.33 (−4.107 to 16.766)	0.234
$CD8^+$ CM $CD57^-PD1^+$	−0.07 (−1.001 to 0.869)	0.890	−0.54 (−1.446 to 0.372)	0.247	+0.77 (−1.242 to 2.776)	0.454
$CD8^+$ CM $KLRG1^-CD57^-$	+1.31 (−1.092 to 3.706)	0.285	−0.28 (−3.130 to 2.575)	0.849	+4.11 (0.093 to 8.130)	**0.045**
**$CD4^+$ lymphocyte subsets**						
$CD4^+$ Effector Memory ($CD28^-MLCD62L^-$)	+3.17 (−1.445 to 7.780)	0.178	+2.60 (−3.346 to 8.553)	0.391	+4.17 (−3.353 to 11.684)	0.278
$CD4^+$ EM $CD57^-KLRG1^-$	−0.35 (−2.462 to 1.761)	0.745	−0.06 (−2.518 to 2.403)	0.964	−0.87 (−4.898 to 3.161)	0.673
$CD4^+$ EM $CD57^-PD1^+$	+0.61 (−2.539 to 3.763)	0.703	+0.35 (−2.722 to 3.432)	0.821	+1.07 (−5.960 to 8.094)	0.766
$CD4^+$ EM $CD57^-PD1^+$	−0.94 (−2.391 to 0.506)	0.202	−1.09 (−3.216 to 1.042)	0.317	−0.69 (−2.173 to 0.800)	0.366
$CD4^+$ EM $KLRG1^-CD57^-$	+1.01 (−0.801 to 2.816)	0.275	+0.49 (−1.443 to 2.426)	0.618	+1.92 (−1.804 to 5.645)	0.312
$CD4^+$ Central Memory ($CD28^-MLCD62L^-$)	−3.18 (−9.711 to 3.347)	0.340	−0.88 (−7.243 to 5.483)	0.786	−7.25 (−21.560 to 7.053)	0.320
$CD4^+$ CM $CD57^-PD1^+$	+0.23 (−1.527 to 1.995)	0.795	+0.42 (−1.631 to 2.474)	0.687	−0.10 (−3.464 to 3.267)	0.954
$CD4^+$ CM $CD57^-KLRG1^-$	−1.42 (−5.361 to 2.517)	0.479	+0.47 (−0.188 to 1.119)	0.163	−4.76 (−15.640 to 6.118)	0.391
$CD4^+$ CM $CD57^-PD1^+$	−0.13 (−0.474 to 0.207)	0.443	−0.05 (−0.421 to 0.327)	0.805	−0.29 (−0.973 to 0.401)	0.415
$CD4^+$ CM $KLRG1^-CD57^-$	+0.23 (−0.312 to 0.779)	0.402	+0.03 (−0.267 to 0.320)	0.861	+0.60 (−0.835 to 2.034)	0.413

Legend: $P < 0.05$ (unadjusted, exploratory). Analyses are exploratory and not pre-specified. No FDR correction applied within strata. One-sample z-test on paired within-subject change-score contrasts (exercise − usual-care period). Values are mean treatment contrast (95% CI) and unadjusted P-value. Women n=22–23, men n=11–13.
